# Disentangling the Sleep-Pain Relationship in Pediatric Chronic Pain: The Mediating Role of Internalizing Mental Health Symptoms

**DOI:** 10.1155/2017/1586921

**Published:** 2017-11-16

**Authors:** Maria Pavlova, Jennifer Ference, Megan Hancock, Melanie Noel

**Affiliations:** ^1^Department of Psychology, University of Calgary, Calgary, AB, Canada; ^2^Alberta Children's Hospital, Calgary, AB, Canada; ^3^Department of Psychology, University of Calgary, Alberta Children's Hospital Research Institute (Behaviour and the Developing Brain Theme), Mathison Centre for Mental Health Research and Education, Calgary, AB, Canada

## Abstract

**Background:**

Pediatric chronic pain often emerges in adolescence and cooccurs with internalizing mental health issues and sleep impairments. Emerging evidence suggests that sleep problems may precede the onset of chronic pain as well as anxiety and depression. Studies conducted in pediatric populations with pain-related chronic illnesses suggest that internalizing mental health symptoms may mediate the sleep-pain relationship; however, this has not been examined in youth with primary pain disorders.

**Objective:**

To examine whether anxiety and depressive symptoms mediated relationships between sleep quality and pain outcomes among youth with chronic pain.

**Methods:**

Participants included 147 youth (66.7% female) aged 8–18 years who were referred to a tertiary-level chronic pain program. At intake, the youth completed psychometrically sound measures of sleep quality, pain intensity, pain interference, and anxiety and depressive symptoms.

**Results:**

As hypothesized, poor sleep quality was associated with increased pain intensity and pain interference, and anxiety and depressive symptoms mediated these sleep-pain relationships.

**Discussion:**

For youth with chronic pain, poor sleep quality may worsen pain through alterations in mood and anxiety; however, prospective research using objective measures is needed. Future research should examine whether targeting sleep and internalizing mental health symptoms in treatments improve pain outcomes in these youth.

## 1. Introduction

Pediatric chronic pain (i.e., pain lasting for three months or longer) tends to emerge in late childhood (recurrent abdominal pain [1]) and early adolescence (musculoskeletal pain [2] and headaches [3]). Prevalence rates of chronic pain are rising (median prevalence rates ranging from 11% to 38%) [4], and longitudinal research suggests that up to 64% of youth with chronic pain will continue to have pain problems into adulthood [5]. Pediatric chronic pain is integrally linked to internalizing mental health symptoms, such as elevated anxiety and depression symptoms (for review, see [6]). Moreover, even when pain resolves by adulthood, its initial occurrence confers risk for lifetime diagnoses of depression and anxiety [7, 8].

In addition to the persistent subjective suffering, sleep impairments are also associated with chronic pain across the lifespan [9], and this cooccurrence is linked to greater functional disability and reduced quality of life [10]. While the sleep-pain relationship was initially posited to be bidirectional [11], a review by Finan and colleagues came to a different conclusion. The majority of reviewed studies were longitudinal and experimental sleep deprivation studies that included adult samples, with three studies investigating the sleep-pain relationship in youth [9]. Overall, Finan et al. found that sleep impairments are a stronger and more reliable predictor of pain than vice versa [9].

Emerging research utilizing microlongitudinal data (i.e., daily assessments of mood and pain) among pediatric pain populations supports this. In youth recovering after a major surgery, poor sleep quality was predictive of higher next-day pain intensity, suggesting that sleep impairments may contribute to persistent postsurgical pain [12]. A similar pattern was found among youth with juvenile idiopathic arthritis (JIA), whose daily reports of worse sleep quality were associated with higher levels of next-day pain intensity [13]. Further, in a sample of youth with chronic pain, longer sleep duration and more minutes awake after sleep onset (i.e., less restorative sleep) were predictive of higher levels of next-day pain intensity [14].

Similar relationships have been reported in epidemiological studies. Sleep problems in childhood (age 10-11 years; [15]) and young adulthood (age 19 years; [16]) were associated with a higher risk of developing pain problems two to three years later [15, 16]. Few studies have empirically examined the mechanisms underlying the sleep-pain relationship in youth; however, negative affect [17] and depressive symptoms [18] have been proposed as potential mechanisms underlying the association between sleep impairments and increased pain intensity. In a sample of youth with chronic pain, lower positive affect and increased negative affect were shown to mediate the relationship between self-reported poor sleep quality and increased functional disability [17]. Further, negative, but not positive, affect mediated the relationship between youth reported poor sleep quality and increased pain intensity [17].

Research suggests that elevated depressive and anxiety symptoms often cooccur with both sleep impairments and chronic pain in youth. Moreover, the earlier that sleep problems emerge in development, the more likely children are to experience symptoms of anxiety and depression by mid-adolescence [19]. Additionally, insomnia at 4.5 years of age that continues to be present by the age of 9 years predicted clinically significant levels of anxiety into early adulthood [20]. Relatedly, depressive symptoms cooccurring with sleep impairments at the age of 5 years were found to be associated with an increased risk of depressive symptoms at the age of 34 [21]. The high cooccurrence of internalizing mental health symptoms and chronic pain in youth has been established (for review, see Vinall et al. [6]), and to account for this high cooccurrence, conceptual models [22] have pointed to sleep impairments (e.g., hyperarousal) as a key mutually maintaining factor. Enhanced understanding of the mechanisms underlying these overlapping, commonly cooccurring difficulties could inform targeted interventions to improve outcomes in these vulnerable youth.

Several conceptual models have been proposed to explain the relationship between sleep and pain in children and adolescents. Lewin and Dahl [11] posit a bidirectional relationship between sleep and pain, where pain is associated with fewer hours of sleep. Decreased sleep duration is related to a range of negative affective and behavioral responses (e.g., decreased attentional control and higher levels of irritability). These consequences, in turn, are associated with increased perception of pain [11]. Valrie et al. [23] extend and complement Lewin and Dahl's conceptual model. Potential mechanisms (e.g., biological factors) contributing to the association between sleep and pain are added as well as functional outcomes (e.g., quality of life). Moreover, this is the first model to propose that negative mood may mediate this relationship [23]. That is, negative mood along with positive emotions, developmental stage, sex, ethnic-cultural, and sociocontextual factors may explain the complex association between pain and sleep in youth. Further, the interplay of these factors influences functional outcomes (e.g., health care use and quality of life) in children and adolescents [23]. A similar conceptual model explains the influence of pediatric chronic fatigue syndrome on sleep impairment [24]. Specifically, an interplay between child's physiological (e.g., hormonal fluctuations), developmental (e.g., developmental stage), sociocultural (e.g., family structure and cultural beliefs), psychological and behavioral (e.g., temperament), and disease-related (e.g., levels of pain and fatigue) factors are thought to influence quality and quantity of sleep [24].

However, the empirical support is limited to pediatric samples of youth with JIA [13], sickle cell disease (SCD) [25], and cancer [18]. Moreover, anxiety symptoms that are common in youth with chronic pain [26] and that are heightened by poor sleep [27] were not included in the model [23] or the recent empirical studies [17]. The current study is the first to examine the mediating roles of anxiety and depressive symptoms in the relationship between sleep quality and pain outcomes, that is, pain intensity and interference in youth with primary pain disorders. We hypothesized that higher anxiety and depressive symptoms would mediate the relationships between poorer sleep quality and worse pain outcomes.

## 2. Materials and Methods

### 2.1. Participants and Setting

159 youth and one of their parents and/or caregivers participated in the current study. They were invited to participate in the study by email or phone prior to their first appointment at a tertiary-level chronic pain program in a pediatric hospital in Canada. Youth aged 8 to 18 years were eligible to participate if they were referred to one of the chronic pain programs for assessment and/or treatment. Youth diagnosed with a developmental disability and/or who did not speak English were excluded from the study.

Data from 147 youth (66.7% female, 13.32 years old (*SD* = 2.59, range 8–18)) who completed at least 80% of the questionnaires were included in the analyses. Youth had been referred to either the abdominal pain (1.4%), complex pain (36.1%), or headache (62.6%) clinic. Sociodemographic information of the sample (i.e., age, sex, ethnicity, and income) is presented in Table 1. Descriptive statistics of the key variables are presented in Table 2. Reported average pain intensity was 5.07/10 (*SD* = 2.34), and average pain interference was 56.52 (*T*-score; *SD* = 9.76). Overall, 43.5% of the youth reported pain to be “always present (intensity varies/the same intensity).” Pain in multiple locations was reported by 62.6% of the youth. On average, pain has been present for 35.17 months (*SD* = 33.98).

### 2.2. Procedure

One to three weeks prior to the first clinical appointment, potential participants were informed about the study over the phone. If they were eligible and interested to participate, online consent forms were sent to youth and one of their parents. Upon providing consent, the youth completed psychometrically sound self-report measures of pain characteristics, pain interference, sleep quality, and anxiety and depressive symptoms using REDCap, a secure online data collection application [28]. Parents reported sociodemographic information. A standard protocol of prompting families to complete the questionnaires up to four times over the course of four weeks was used. The institutional Research Ethics Board (REB) approved the study.

### 2.3. Measures

#### 2.3.1. Demographics

Parents reported on their child's age, sex, and ethnicity, their relationship to the child, and household income.

#### 2.3.2. Pain Characteristics

Youth completed the valid and reliable pain questionnaire [29]. Youth reported their pain location using a validated body map [30] (Table 1). They also reported the duration of their pain problem (in months) and pain frequency using a 5-point Likert scale ranging from “rarely present pain (occurs every few days or weeks)” to “always present (always the same intensity).”

#### 2.3.3. Sleep Quality

The revised Adolescent Sleep-Wake Scale (rASWS) was administered to assess sleep quality in the past seven days on a 6-point Likert scale (anchors: 1 = “never” and 6 = “always”) [31]. Ten empirically derived items of the original ASWS [32] were averaged to yield three subscale scores, that is, Falling Asleep and Reinitiating Sleep, Returning to Wakefulness, and Going to Bed. A total score (i.e., a mean of three subscales) indicated the overall quality of sleep, with higher scores representing better sleep quality. The rASWS has been validated for use in youth with a variety of health conditions, including youth with chronic pain [31].

#### 2.3.4. Pain Outcomes—Intensity and Interference

Youth completed the Patient-Reported Outcomes Measurement Information System (PROMIS) Pediatric Profile-25 that is a part of the NIH assessment toolbox [33]. The PROMIS instruments have been rigorously developed to assess mental and physical health in youth and adults and have been validated for use in pediatric samples with chronic pain [34]. Item response theory and the associated calibration of the individual items allowed for creation of brief, sensitive-to-change forms with a smaller standard error of measurement [34]. The Pain Interference subscale assessed how much pain interfered with youth's everyday activities in the past seven days using a 5-point Likert scale (anchors: 0 = “never” and 4 = “almost always”) (e.g., “It was hard for me to walk when I had pain”). This scale is composed of four items that yield a standardized *T*-score used in the analyses. Average pain intensity in the past 7 days was reported using an 11-point Numeric Rating Scale item from the PROMIS Pediatric Profile-25 (anchors: 0 = “no pain” and 10 = “worst pain you can think of”). The PROMIS measures demonstrated good construct validity (intercept and slope ≥ 0.98 [34]) and internal consistency (pain interference, 4 items *α* = 0.85) in a sample of youth with chronic pain.

#### 2.3.5. Anxiety and Depressive Symptoms

Anxiety and depressive symptoms were assessed using the PROMIS Pediatric Profile-25 Anxiety and Depression subscales [33]. Participants reported if they experienced any of the symptoms (e.g., “I felt everything in my life went wrong” and “I felt like something awful might happen”) in the past 7 days using a 5-point Likert scale (anchors: 0 = “never” and 4 = “almost always”). The summed scores of each subscale were transformed into standardized *T*-scores for the analyses. The subscales have demonstrated good construct validity (intercept and slope ≥ 0.93 [34]) and excellent internal consistency (depressive symptoms, 4 items, *α* = 0.91; anxiety symptoms, 4 items, *α* = 0.90). The subscales have been validated in youth with chronic pain [34]. Their brevity reduces participant burden in real clinical settings while still providing psychometrically sound self-report assessment of core constructs in chronic pain [34].

### 2.4. Statistical Analyses

Statistical analyses were conducted using the Statistical Package for the Social Sciences (SPSS), version 24. Prorated scores were used for missing data (less than 20% within each subscale) [35]. Participants who completed less than 80% on each subscale (*n* = 12) were excluded. Demographic characteristics of the sample were reported using descriptive statistics. Frequency statistics were used for categorical variables (youth's sex, household income, and pain location and frequency). Means and standard deviations were reported for all key continuous variables. *T*-tests were used to examine differences in key variables as a function of main sociodemographic variables, that is, youth's age and sex. Bivariate correlations between key variables were conducted to justify inclusion in the mediation models. Specifically, to be included in the analyses, sleep quality had to be significantly correlated with anxiety and/or depressive symptoms. The latter, in turn, had to be significantly associated with pain outcomes, that is, intensity and interference. The relationship between sleep quality and pain outcomes did not have to be significant, but it was tested. Mediation analyses were conducted using the Preacher and Hayes' PROCESS macro for SPSS [36].

**Figure 1:**
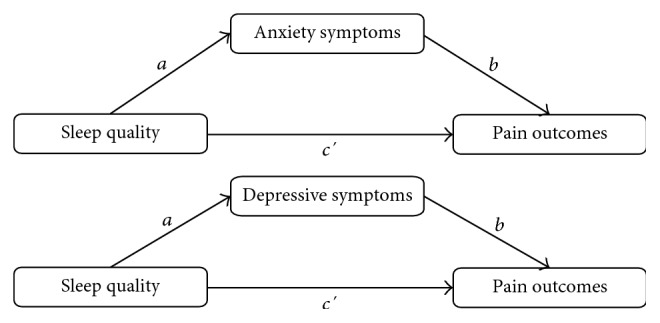
Proposed mediation models.

Based on the correlational analyses, four mediation models (Figure 1) were tested to examine the associations between youth sleep quality and pain outcomes, that is, pain intensity and pain interference. In the first two models, anxiety symptoms were included as a mediator. In the remaining two models, depressive symptoms were included as a mediator. The total effect of sleep quality on pain outcomes (weight *c*) consisted of a direct effect of sleep quality on pain outcomes (weight *c*′) and an indirect effect of sleep quality on pain outcomes through the mediator, anxiety, or depressive symptoms (weight *ab*). In Figure 1, weight *a* denotes the effect of sleep quality on anxiety or depressive symptoms, weight *b* denotes the effect of anxiety or depressive symptoms on pain outcomes.

The main indication of mediation is the presence of an indirect effect, that is, a significant contribution of the mediator to the relationship between the independent and dependent variables. Confidence intervals that do not contain zero suggest with 95% confidence that the indirect effect is not zero [37]. Mediation is established if the confidence interval does not contain zero. To maximize robustness of the results, bootstrapping with 10,000 samples, a preferable method of testing indirect effects of mediation [36], was used in testing the mediation models.

## 3. Results

### 3.1. Descriptive Statistics

On average, youth sleep quality was 3.40/6.00 (*SD* = 0.80). Internalizing symptoms averaged 49.05 for anxiety symptoms (*SD* = 11.86) and 49.72 for depressive symptoms (*SD* = 11.12). Girls reported higher levels of anxiety symptoms (*t*(142) = 2.19, *p*=0.030), pain interference (*t*(75) = 3.11, *p*=0.003), and worse quality of sleep (*t*(145) = −2.22, *p*=0.028), as compared to boys. Age was significantly correlated with depressive symptoms with older youth reporting significantly higher levels of depressive symptoms (*r* = 0.29, *p<0.001*). Therefore, age and sex were entered as covariates in all tested mediation models.

### 3.2. Correlational Analyses

Bivariate correlations between key variables and outcomes are presented in Table 3. Poor sleep quality was associated with higher levels of anxiety (*r* = −0.40, *p<0.001*) and depressive (*r* = −0.39, *p<0.001*) symptoms as well as higher pain intensity (*r* = −0.20, *p*=0.016) and pain interference (*r* = −0.43, *p<0.001*). Higher anxiety and depressive symptoms were correlated with higher pain intensity (*r* = 0.27, *p*=0.001; *r* = 0.22, *p*=0.008) and interference (*r* = 0.48, *p* < 0.001; *r* = 0.41, *p<0.001*).

### 3.3. Mediation Analyses

#### 3.3.1. Anxiety Symptoms as a Mediator in the Relationship between Sleep Quality and Pain Outcomes

Two separate models were tested to investigate whether anxiety symptoms mediated the effect of sleep quality on pain outcomes (i.e., pain interference and pain intensity). Youth's age and sex were included as covariates (Table 4 for regression coefficients and indirect effects). As hypothesized, anxiety symptoms were a partial mediator of the relationships between sleep quality and pain interference as well as sleep quality and pain intensity, over and beyond the influences of child age and sex (anxiety symptoms and pain intensity: *n* = 144, *PE* = −0.25, *SE* = 0.11 (*CI*_BCa_ = −0.52 to −0.07); anxiety symptoms and pain interference: *n* = 142, *PE* = −1.58, *SE* = 0.49 (*CI*_BCa_ = −2.75 to −0.77)).

#### 3.3.2. Depressive Symptoms as a Mediator in the Relationship between Sleep Quality and Pain Outcomes

Similarly, two separate models were tested to examine whether depressive symptoms mediated the effect of sleep quality on pain intensity and pain interference. Consistent with hypotheses, depressive symptoms partially mediated the associations between sleep quality and pain intensity as well as sleep quality and pain interference, over and beyond the influences of child age and sex (depressive symptoms and pain intensity: *n* = 147, *PE* = −0.22, *SE* = 0.11 (*CI*_BCa_ = −0.46 to −0.02); depressive symptoms and pain interference: *n* = 145, *PE* = −1.33, *SE* = 0.46 (*CI*_BCa_ = −2.40 to −0.57)).

## 4. Discussion

The current study is the first to examine anxiety and depressive symptoms as mediators in the relationship between sleep quality and pain outcomes (intensity and interference) in youth with primary pain disorders. As hypothesized, both anxiety and depressive symptoms partially mediated these relationships. This suggests that internalizing mental health symptoms may be a mechanism or a process underlying the relationship between sleep quality and chronic pain outcomes in youth and should be considered both theoretically and clinically.

Our findings support and complement Valrie et al.'s theoretical model that hypothesized mood as one of the factors influencing the sleep-pain relationship in pediatric populations with pain-related chronic illness (juvenile idiopathic arthritis and sickle cell disease) and primary pain disorders [23]. The model assumes a bidirectional relationship between sleep and pain perception, which, in turn, affects health-related quality of life, functional disability, and health care utilization [23]. In addition to mood alterations, physiological and biological factors were also posited to influence the sleep-pain relationship. The current findings are also consistent with the existing research in pediatric populations with pain-related chronic illness (e.g., JIA, oncology/hematology-related illness, and SCD) [13, 18, 25]. Moreover, our findings extend this previous work by providing evidence that depressive symptoms mediate the sleep-pain relationship in a pediatric sample with primary pain disorders. The mediating role of depressive symptoms in sleep quality-pain outcomes relationship is also in line with the recent research showing that negative affect mediated the relationship between poor sleep and functional disability and pain intensity in youth with chronic pain [17]. In the current study, anxiety symptoms were also found to mediate the association between sleep quality and pain outcomes. Thus, anxiety symptoms could be added to future revisions of Valrie et al.'s conceptual model [23] as an additional mechanism that in part explains why sleep impairments and pain problems cooccur in youth.

The current findings also support the conceptual model of pediatric sleep and pain proposed by Lewin and Dahl [11]. Specifically, their model suggests that acute and chronic pain is associated with decreased sleep duration in children and adolescents. Sleep impairments, in turn, are thought to result in higher levels of fatigue, irritability and affective disturbance, as well as decreases in attentional control and positive coping behaviours [11]. These consequences of sleep impairments were posited to heighten pain perception, thus creating a vicious, self-maintaining sleep-pain cycle. Depressive and anxiety symptoms may independently contribute to this sleep-pain cycle through a range of cognitive and affective mechanisms. For example, elevations in anxiety symptoms are linked to higher presleep arousal, which may delay sleep onset and is associated with poorer sleep quality [38]. Moreover, elevated anxiety symptoms may also increase hypervigilance [39] and attentional biases (e.g., increased tendency to selectively attend to pain) which, in turn, disturb sleep [11]. The proposed sequelae of sleep impairments (e.g., increased irritability and alterations in emotion [11]) overlap with depressive symptoms (anhedonia and irritability) and may, in part, explain the contribution of negative mood to the sleep-pain relationship. The findings of experimental studies examining sleep deprivation in adult clinical samples converge to suggest that sleep impairments may lead to both altered pain perception (e.g., hyperalgesia [40] and elevated pain sensitivity [41]) and higher levels of depressed mood [42].

Beyond internalizing mental health symptoms, it is important to consider other potential factors contributing to the sleep-pain relationship. Neurobiological mechanisms are likely to play a key role. For example, sleep architecture may explain the association between sleep impairments and pain. Disrupted slow wave sleep (SWS) has been linked to lower pain thresholds in experimental pain studies with healthy adults (for review see [43]). Reductions in SWS have also been demonstrated in adults with chronic pain [44] and children with pain-related chronic illness (JIA) [45]. Moreover, brain structures that regulate sleep (e.g., reticular nucleus of thalamus and midbrain periaqueductal gray) are also involved in pain modulation [43] and chronic pain maintenance (e.g., recurrent migraines [46]). Another potential pathway could involve neurotransmitter networks. Finan et al. [9] summarized literature supporting the role of the mesolimbic dopamine system in influencing linkages between insomnia, chronic pain, and depression. Overall, existing research exploring neurobiological mechanisms of sleep-pain relationship is limited to adult populations. Given rapid changes in the developing brain and neurocircuitry during the period of adolescence, future research should examine the neurobiological pathways underlying the sleep-pain relationship in pediatric populations.

In addition to internalizing mental health symptoms, future research should also examine the role of *positive* affect on the relationship between sleep impairments and chronic pain outcomes in youth. In a recent study, positive affect was found to mediate the association between sleep quality and functional disability (but not pain intensity) in a sample of youth with chronic pain [17]. Positive affect may facilitate engagement in positive coping behaviours (e.g., attending away from pain sensations), which could lead to attenuation of sleep-pain cycle. To date, the few studies examining positive affect [17, 47] have used the Positive and Negative Affect Schedule (PANAS), an instrument targeted to measure subjective distress or absence thereof (negative affect) and pleasurable engagement with the environment (positive affect) [48]. Moreover, while it is crucial to examine the role of positive affect, it would be beneficial to consider other resilience factors implicated in the chronic pain experience (e.g., optimism [49]) as they may also serve to buffer the impact of poor sleep on pain and functioning.

The findings of our study should be viewed in light of limitations that may be addressed in future research. First, the cross-sectional nature of the current study prevents inferences about the directionality of the relationships between our variables. Mediation analyses are ideally used in longitudinal research designs as it allows a more rigorous investigation of underlying mechanisms [50]. Given that our study is cross-sectional in nature, this is a methodological limitation and future longitudinal work is needed. However, accumulating basic and clinical investigations provide compelling evidence that sleep problems more strongly drive increases in pain [9] and internalizing mental health conditions [51], than vice versa. Longitudinal studies assessing mood, anxiety, sleep, and pain on a daily basis are needed to examine fluctuations and dynamic relationships between these factors in more ecologically valid settings over time. Existing microlongitudinal studies have captured daily relationships between mood and pain [52] or pain levels and sleep [14]; however, our results suggest that investigations integrating all three factors/processes are needed.

Second, the current study assessed anxiety and depression at a global symptom, rather than a diagnostic, level; therefore, the applicability of findings to youth with clinical diagnoses of anxiety and depression or particular types of anxiety (e.g., social and generalized) is not known. Nevertheless, given that this relationship was present among youth with subclinical symptom presentations, it is plausible that these relationships would be even stronger in the context of clinically diagnosed anxiety and/or depressive disorders. Third, we did not assess positive affect, a construct that is independent of depressive symptoms and that has been shown to buffer the negative associations between sleep impairments and pain outcomes [9, 13]. Future studies should include measures of positive affect to examine its unique contributions to the sleep-pain associations in youth with chronic pain. Finally, the sample was mostly white with a reported annual income greater than $90,000, thereby limiting the generalizability to more diverse populations of youth with chronic pain.

Our findings emphasize the importance of addressing internalizing mental health symptoms and sleep impairments in the treatment of pediatric chronic pain given their negative influence on pain outcomes. Although sleep hygiene is sometimes included in cognitive-behaviour therapy (CBT) interventions for chronic pain [53], mental health issues are often not formally addressed. Improving sleep and internalizing mental health symptoms in more integrated chronic pain interventions could improve treatment outcomes in these youth. Cognitive-behaviour therapy for insomnia (CBT-I) has been used in adult [51] and pediatric populations [54] with a variety of comorbid mental health conditions to improve sleep and comorbid psychiatric or pain conditions. Core elements of these treatments include sleep restriction, stimulus control, and sleep hygiene [55]. Preliminary results of a trial of a 4-session CBT-I intervention for youth with insomnia and a comorbid psychological (e.g., depression) or physical (e.g., asthma, chronic pain) diagnosis demonstrated improvements in sleep quality as well as in health-related quality of life [54]. Some trials of CBT-I in adult populations with pain-related chronic illnesses (e.g., osteoarthritis) have shown improvements in pain [56], whereas others have not [57]. Limited evidence supports efficacy of CBT with sleep-specific components for pain outcomes in pediatric populations with pain-related chronic conditions (e.g., fibromyalgia [53]). The evidence for the influence of CBT-I interventions on improving mental health outcomes is relatively stronger. Given that poor sleep has been reported to impede the progress of CBT treatments for both chronic pain and depression in children and adolescents [58, 59], the findings of the present study provide additional rationale for addressing sleep issues to potentially reduce elevations in internalizing mental health symptoms and improve pain and functioning. In the context of chronic pain, it is likely that approaches that integrate targeting mental health, sleep, and pain will be most effective.

In conclusion, this study was the first to demonstrate that elevated anxiety and depressive symptoms partially mediate the relationship between poor sleep quality and higher levels of pain intensity and pain interference among youth with primary pain disorders. The findings point to potential affective mechanisms underlying the highly comorbid relationship between pediatric chronic pain and sleep problems and provide support for, and extend, existing conceptual models of sleep and pain in youth. The study also highlights a critical need for addressing internalizing mental health symptoms and sleep within the context of pediatric chronic pain. Future longitudinal studies are needed to further investigate these proposed mechanisms.

## Figures and Tables

**Table 1:** Sociodemographic characteristics of the sample.

Sociodemographics	*N* = 147
Child's age (*M* years, *SD*)	13.32 (2.59)
Child's sex (% female)	66.7
Parent's sex (% female)	92.5
Relationship to the child (%)	
Biological parent	97.9
Adoptive parent	1.4
Legal guardian	0.7
Child's ethnicity (%)	
White (Caucasian)	81.4
Two or more ethnicities	9.0
Latin American	3.5
Arab/West Asian	1.4
Other	2.8
Do not want to answer	0.7
Household income (%)	
<$10,000–$29,999	4.3
$30,000–$59,999	9.3
$60,000–$89,999	9.3
More than $90,000	54.3
Do not want to answer	22.9
Pain locations (%)	
Multiple locations	62.6
Single location	37.4
Head	18.4
Limb	8.8
No location reported	4.8
Abdomen	2.7
Face	0.7
Chest	0.7
Groin	0.7
Hip	0.7

**Table 2:** Descriptive statistics for key variables.

Variable	*M* (*SD*)
Sleep quality (rASWS), total	3.40 (0.80)
Anxiety symptoms (PROMIS), *T*-score	49.05 (11.86)
Depressive symptoms (PROMIS), *T*-score	49.72 (11.12)
Pain intensity (PROMIS), total	5.07 (2.34)
Pain interference (PROMIS), *T*-score	56.52 (9.76)

*Note*. rASWS = revised Adolescent Sleep-Wake Scale; PROMIS = Patient-Reported Outcomes Measurement Information System. Means of prorated total scores are displayed for the rASWS. Means of *T*-scores are displayed for pain interference and depressive and anxiety symptoms.

**Table 3:** Correlations among key variables.

Variable	1	2	3	4	5
(1) rASWS, total	1	−0.40^∗∗∗^	−0.39^∗∗∗^	−0.20^∗^	−0.43^∗∗∗^
(2) PROMIS—anxiety symptoms, *T*-score	—	1	0.71^∗∗∗^	0.27^∗∗^	0.48^∗∗∗^
(3) PROMIS—depressive symptoms, *T*-score	—	—	1	0.22^∗∗^	0.41^∗∗∗^
(4) PROMIS—pain intensity, *T*-score	—	—	—	1	0.54^∗∗∗^
(5) PROMIS—pain interference, *T*-score	—	—	—	—	1

*Note*. ^∗^*p<0.05*, ^∗∗^*p<0.01*, ^∗∗∗^*p<0.001* two-tailed test. rASWS = revised Adolescent Sleep-Wake Scale; PROMIS = Patient-Reported Outcomes Measurement Information System.

**Table 4:** Unstandardized coefficients and indirect effect sizes.

Model	*b*	*SE*	*t*	*p*	*CI*_BCa_ (LL)	*CI*_BCa_ (UL)
*Pain intensity*						
Sleep quality → anxiety symptoms (*a*)	−5.60	1.16	−4.84	<0.001	−7.89	−3.32
Anxiety symptoms → pain intensity (*b*)	0.04	0.02	2.48	0.014	0.01	0.08
Sleep quality → pain intensity (*c*′)	−0.30	0.26	−1.15	0.254	−0.82	0.22
Sleep quality → anxiety symptoms → pain intensity (a ∗ b)	−0.25	0.11	—	—	−0.52	−0.07
*Pain interference*						
Sleep quality → anxiety symptoms (*a*)	−5.73	1.16	−4.94	<0.001	−8.02	−3.44
Anxiety symptoms → pain interference (*b*)	0.28	0.06	4.28	<0.001	0.15	0.40
Sleep quality → pain interference (*c*′)	−3.20	0.95	−3.37	0.001	−5.09	−1.33
Sleep quality → anxiety symptoms → pain interference (a ∗ b)	−1.58	0.49	—	—	−2.75	−0.77
*Pain intensity*						
Sleep quality → depressive symptoms (*a*)	−5.37	1.04	−5.15	<0.001	−7.44	−3.31
Depressive symptoms → pain intensity (*b*)	0.04	0.02	2.08	0.040	0.002	0.08
Sleep quality → pain intensity (*c*′)	−0.32	0.26	−1.21	0.228	−0.84	0.20
Sleep quality → depressive symptoms → pain intensity (a ∗ b)	−0.22	0.11	—	—	−0.46	−0.02
*Pain interference*						
Sleep quality → depressive symptoms (*a*)	−5.40	1.05	−5.13	<0.001	−7.48	−3.32
Depressive symptoms → Pain interference (*b*)	0.25	0.07	3.50	0.001	0.11	0.39
Sleep quality → pain interference (*c*′)	−3.49	0.96	−3.63	0.0004	−5.39	−1.59
Sleep quality → depressive symptoms → pain interference (a ∗ b)	−1.33	0.46	—	—	−2.40	−0.57

*Note*. *N* for analyses is 144 cases for pain intensity-anxiety symptoms, 142 cases for pain interference-anxiety symptoms, 147 cases for pain intensity-depressive symptoms, and 145 cases for pain interference-depressive symptoms models. Sleep quality (rASWS, total score) is the independent variable (IV) in all models. Anxiety or depressive symptoms (PROMIS Anxiety and Depression subscales, *T*-scores; M); pain intensity (NRS; DV1); and pain interference (PROMIS Pain Interference subscale, *T*-score; DV2) are the outcome variables. *CI*_BCa_ (LL) = lower limit of a 95% confidence interval; *CI*_BCa_ (UL) = upper limit. Analyses are controlling for youth's age and sex.
